# Unna’s Ichthyol Ointment for Profuse Sweating of the Feet

**Published:** 1898-02

**Authors:** 


**Unna's Ichthyol Ointment for Profuse Sweating of the Feet.**—The formula is given as follows in the *Gazette hebdomadaire de medicine et de chirurgie* for October 14th:

| | | |
|---|---|---|
| ℞ | Ichthyol | 25 parts. |
| | Water | 15 parts. |
| | Lanolin | 15 parts. |

M.

---

**Veterinary Surgeon** (to his new assistant)—"You must take this tube, Pat, fill it with this powder, insert it in the horse's mouth and give a quick, sharp blow."

Vet. (ten minutes later).—"What's the trouble, Pat?"

Pat.—"Troth, sir, the horse blowed first."—*Colorado Medical Journal.*

